# Wild jackdaws’ reproductive success and their offspring’s stress hormones are connected to provisioning rate and brood size, not to parental neophobia

**DOI:** 10.1016/j.ygcen.2016.11.006

**Published:** 2017-03-01

**Authors:** Alison L. Greggor, Karen A. Spencer, Nicola S. Clayton, Alex Thornton

**Affiliations:** a Department of Psychology, University of Cambridge, UK; b Department of Biological Sciences, Dartmouth College, USA; c School of Psychology and Neuroscience, University of St. Andrews, UK; d Department of Biosciences, University of Exeter, Penryn, UK

**Keywords:** Brood size, Corticosterone, Corvidae, Developmental stress, Fitness, Neophobia

## Abstract

Many species show individual variation in neophobia and stress hormones, but the causes and consequences of this variation in the wild are unclear. Variation in neophobia levels could affect the number of offspring animals produce, and more subtly influence the rearing environment and offspring development. Nutritional deficits during development can elevate levels of stress hormones that trigger long-term effects on learning, memory, and survival. Therefore measuring offspring stress hormone levels, such as corticosterone (CORT), helps determine if parental neophobia influences the condition and developmental trajectory of young. As a highly neophobic species, jackdaws (*Corvus monedula*) are excellent for exploring the potential effects of parental neophobia on developing offspring. We investigated if neophobic responses, alongside known drivers of fitness, influence nest success and offspring hormone responses in wild breeding jackdaws. Despite its consistency across the breeding season, and suggestions in the literature that it should have importance for reproductive fitness, parental neophobia did not predict nest success, provisioning rates or offspring hormone levels. Instead, sibling competition and poor parental care contributed to natural variation in stress responses. Parents with lower provisioning rates fledged fewer chicks, chicks from larger broods had elevated baseline CORT levels, and chicks with later hatching dates showed higher stress-induced CORT levels. Since CORT levels may influence the expression of adult neophobia, variation in juvenile stress responses could explain the development and maintenance of neophobic variation within the adult population.

## Highlights

• Parental neophobia does not predict fitness or offspring CORT in wild jackdaws.
• Parents with lower provisioning rates fledge fewer chicks.
• Chicks from larger broods have higher baseline CORT levels.
• Chicks with later hatching dates show higher stress-induced CORT levels.
• The fitness-related and ecological consequences of neophobia are still unclear.

## 1 Introduction

Neophobia, or the fear of novelty, allows animals to avoid unknown danger, but may also prevent the exploitation of new resources (Greenberg and Mettke-Hofmann, 2001). Individuals who express elevated neophobia may be more likely to survive when predation pressure is high (Ferrari et al., 2015), but they may be at a disadvantage when gathering resources in variable environments, since high neophobia can inhibit behavioral innovation (Benson-Amram and Holekamp, 2012, Greenberg, 2003). Although many species are thought to show consistent individual variation in neophobia (Réale et al., 2007), the consequences of this variation in the wild are poorly understood. Behaviors such as neophobia that can be classified as responses to change or uncertainty in the environment, and are consistent at least within seasons, are proposed to have important consequences for individual fitness (Dall et al., 2004). Meta-analyses reveal that less fearful, or “bolder” individuals typically have higher reproductive success (Smith and Blumstein, 2008). However, the majority of evidence for connections between object neophobia and fitness come from studies in which behavioral measures and/or subsequent reproductive success were assessed in captivity (Bremner-Harrison et al., 2004, Janczak et al., 2003, Korhnonen et al., 2002, Korhonen and Niemela, 1996, Korhonen et al., 2001). To our knowledge, only one study has found correlations between neophobia and reproductive output in the wild, reporting that neophobic great tits (*Parus major*) had higher occurrences of nest failure than their less fearful conspecifics (Vrublevska et al., 2015). Direct measures of the impact of neophobia in the wild are rare (although see Schuett et al., 2012). Moreover, even if parental neophobia levels do not impact the gross number of offspring produced, they could have more subtle influences on the rearing environment and the later development of offspring if they prevent the parent from providing adequate or predictable food sources. However, connections between neophobia, foraging ability, and their impact on offspring quality have yet to be tested.

If neophobia levels affect parents’ ability to forage for their young, then parental neophobia would be expected to predict measures that indicate developmental stress and offspring quality. Although stress can be caused by numerous factors, such as food deprivation (Pravosudov and Kitaysky, 2006), disrupted maternal care (Banerjee et al., 2012), and acute stressful events (Jacobson-Pick and Richter-Levin, 2010), elevated stress hormones are a common mechanism by which stress impacts a growing organism. One stress hormone, corticosterone (CORT), naturally circulates at a baseline level in the blood to regulate physiological processes such as animals’ circadian rhythm. CORT levels also increase dramatically after a stressful event to prime animals for a “fight or flight” response (Romero, 2004, Sapolsky et al., 2000). Therefore, elevated levels of baseline CORT may serve as a marker of ongoing or developmental stress, and exaggerated levels of CORT during stressful events can indicate the magnitude of an individual’s fear response (Romero, 2004). Together, the long-term combination of routine CORT release and release during acute stressors determines an individuals’ allostatic load, i.e. the wear and tear from cumulative stress. A high allostatic load increases the potential for hormone dysregulation (Romero et al., 2009), and may affect their ability to respond to environmental changes (Wingfield, 2013).

Although short-term increases in CORT can help an individual survive a life threatening event, experiencing chronically elevated levels of CORT during development can have long-term effects ranging from impairments in brain structure (Welberg and Seckl, 2001), to reductions in life expectancy (Monaghan et al., 2012), and implications for immune function (Kriengwatana et al., 2013). Long term stress can also decrease the sensitivity of glucocorticoid receptors present in the brain (Banerjee et al., 2012, Hodgson et al., 2007) which potentially modifies the negative feedback loops of stress hormone expression (Romero, 2004, Zimmer et al., 2013). Therefore responses to stress and levels of circulating CORT are often considered stable traits (Evans et al., 2006, Jenkins et al., 2014, Kralj-Fišer et al., 2007; although see Ouyang et al., 2011), and have been suggested to drive individual differences in avian temperament or personality (Baugh et al., 2012, Cockrem, 2007, Moretz et al., 2007). Although many species show individual and population level variation in stress hormone expression (e.g. Cockrem and Silverin, 2002, Grunst et al., 2014, Liebl and Martin, 2012) that can be heritable (Evans et al., 2006), the factors driving this variation differ depending on the species (e.g. food deprivation in western scrub jays, *Aphelocoma californica* Pravosudov and Kitaysky, 2006; sibling competition in barn swallows, *Hirundo rustica* Saino et al., 2003). Therefore the drivers of stress hormone variation are not well understood, despite their potentially far-reaching consequences for development and behavior.

One species that could help disentangle the relationship between neophobia, fitness and offspring rearing environment is the jackdaw (*Corvus monedula*). Like other members of the corvid family, jackdaws are known for having high levels of neophobia in comparison to other species (Greenberg and Mettke-Hofmann, 2001, Greggor et al., 2016a). Individual variation in neophobia and other forms of wariness have been documented in jackdaws (Greggor et al., 2016c, Schuett et al., 2012), but the consequences of their comparatively high neophobia are still unclear. Although a previous study on jackdaws found no relationship between a single object neophobia measure and the number of chicks produced in one season (Schuett et al., 2012), it is unclear if the neophobia measure was repeatable within the season. Also it is unclear whether or not neophobia would have correlated with nest success had feeding rate—the principal determinant of jackdaw reproductive success (Henderson and Hart, 1993)—been accounted for. Since feeding rate has also been implicated in influencing connections between fitness and responses to novelty in other contexts (e.g. towards a novel environment, at least in females; Mutzel et al., 2013) it could be crucial for determining the origin of neophobia-related fitness effects in jackdaws. Finally, even if parents’ neophobia does not influence the net number of chicks they produce per season, it could still broadly impact the quality of the rearing environment and the subsequent physiological stress responses of their offspring. Such influences are critical to determining the potential costs and benefits of neophobic behavior because the effects of developmental impairment could occur after chicks fledge.

We examined the connections between parental neophobia levels, provisioning rates, and breeding success (i.e. fledgling number and quality) in wild breeding jackdaws. We then looked at a subset of nests to assess whether these factors influenced chicks’ baseline and stress-induced CORT expression, when other potential influences on CORT such as brood size were considered (Saino et al., 2003). We predicted, similar to what Schuett et al. (2012) reported, that parents’ neophobia would not correlate with fledgling number. Instead neophobic variation could influence offspring in other, less direct ways by reducing provisioning rates to an extent that impacts fledging chicks’ body condition or alters baseline circulating CORT and juveniles’ propensity to mount a stress-induced hormone response. Therefore even if parents’ neophobia does not directly impact chicks’ survival in the nest, it could have other long-term impacts on offspring development that would explain selection for or against neophobic behavior.

## 2 Methods

### 2.1 Study sites

The study site was situated in forested areas surrounding Madingley Village, Cambridgeshire, UK. Nest boxes were erected on private University of Cambridge land that remains largely undisturbed. During the breeding season of 2013 we tested 68 jackdaw nest boxes on neophobia, and measured CORT levels in 58 chicks from 34 of these boxes. Boxes were clustered into 14 colonies within the study site. Boxes were placed on trees 3–4 m off the ground, such that chicks could be accessed via a large extendable ladder.

The study site was monitored throughout the breeding season. Since jackdaws only have one brood per season, even if their nest fails (Röell, 1978), our monitoring captured the reproductive success of each pair for that year. Laying and hatch dates were determined by daily nest checks. Since jackdaw nests hatch asynchronously, we checked nests daily until all eggs hatched or until several days had lapsed with no new chicks emerging. After all viable eggs hatched, boxes were monitored at least three times a week. Daily checks resumed again at day 28 as the fledging period approached (day 32–34), to provide information on nestling mortality and nest failure. Chicks that died due to starvation could be easily identified because jackdaw parents are unable to remove them from the box once they reach about 10 days of age. We deemed the nest to have fledged once all chicks vacated the box. All nest disturbances were conducted under a Natural England License (20130067 to A.L.G.), blood sampling under Home Office permits (PIL 70/24971 to A.L.G, PPL to A.T. 80/2371) and ringing under British Trust for Ornithology license (No. C6079, C5752, C5746).

### 2.2 Experimental protocol and blood sampling

**Fig. 1:**
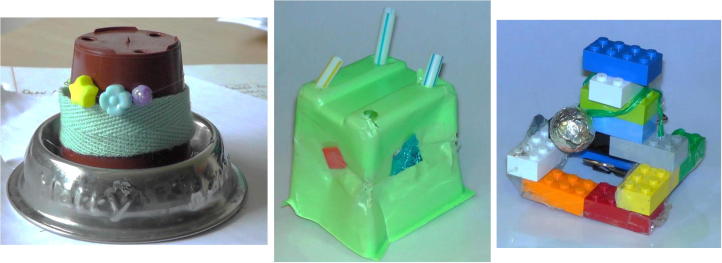
Novel objects used on nest boxes.

Three novel objects were constructed out of bright, man-made materials, without elements that resembled eyes, or an animal shape (see Fig. 1). Exact replicas of each object were constructed to allow for concurrent testing across nest box colonies within the study site. Each nest box was tested with two objects over the course of the study. No box received the same object more than once. Each object contained the same large clip to attach it to the nest-box’s platform via an extendable pole while minimizing disturbance (see Supplementary Fig. S1). Boxes were tested between the hours of 7:00 and 18:30. Each box was tested twice; once during the first half (6.27 ± 0.29 days since hatching) of hatchling development, and once during the second half (20.75 ± 0.36 days since hatching). Each test was paired with a control trial at the same nestbox that was randomly assigned to occur either the prior or following day at the same time. For each test, the video camera (Panasonic HC-V130) was set up, and the object was presented at the hole of the box to flush out adults that may not have heard the experimenter approach. The object was then placed onto the platform with the extendable pole, and the experimenter left the vicinity of the nest site. Control trials were set up identically to tests, except that no object was placed on the pole when it was presented at the box hole. The order of control vs. test trials, and the specific object used were determined using a random number generator prior to the beginning of the study. No two boxes in the same colony were tested concurrently. Each trial lasted 90 min and was video recorded from a camouflaged tripod.

Blood sampling took place in conjunction with nestling ringing on the 25th day of life of each box’s oldest chick. Boxes were approached quietly to avoid disturbing the chicks ahead of sampling. All sampling was conducted at least 2 h after sunrise, and 2 h before sunset. Baseline samples (100 μl) for all chicks within the nest were collected within or as close to three minutes as possible (Romero and Reed, 2005) of the ladder touching the tree of the nest box (mean 2.34 ± 0.59 min). Stress-induced samples were collected for each individual 10 min after their baseline sampling time. Sample collection times were noted to the nearest second. Between samples, chicks’ wing and tarsus length were measured using calipers (to the nearest 0.1 mm) and weight was recorded (to the nearest 0.1 g using an electronic balance). The chicks were returned to their nest after they had been processed. The blood was immediately put on ice, and spun in a centrifuge within 3 h of collection. The plasma was separated from the rest of the blood sample and frozen at −80 °C until it was analyzed. The remaining red blood cells were diluted with 1 ml of ethanol and used for molecular sexing analysis with PCR.

CORT hormone concentrations were determined from plasma samples through direct radio-immunoassay (see Spencer et al., 2009). Aliquots of jackdaw plasma (∼20 μl) and three sets of standard chicken plasma with known CORT concentration were combined with 25 μl of radiolabeled CORT and left to rest for 1hr. The samples were extracted with 1 ml ether, and reconstituted with 300 μl assay buffer. Samples were run in duplicates, under a standard RIA procedure (e.g. Wingfield et al., 1992), with primary antibody AB-ine880, supplied by Antibodies Online. Calculations of hormone concentrations were corrected for variation in initial aliquot volume and individual recoveries; the mean percent recovery was 96.3 ± 9.4. Samples were run in three assays, and samples from siblings and nest box colonies were randomized across assays. Individuals’ baseline and stress-induced measures were always run in the same assay. Intra-assay coefficients of variation were 5.0%, 5.2%, and 2.3% and the inter-assay CV was 21.4%. The detection limit was 0.08 ng/ml.

### 2.3 Behavioral data

Neophobia was measured from video recordings, and defined as the time elapsed between the beginning of the trial to when the first bird entered the nest (e.g. Schuett et al., 2012). The initial entrance time was similarly noted for control trials, as was the number of entrances that occurred during the remainder of the 90 min control trial. Neophobia scores were determined by averaging both test entrance times. Meanwhile control scores were determined by the two control entrance times. If the nest failed before the second pair of test and control tirals was conducted, the first set of entrance times were used (see Section 3.1 for details). Feeding rate was determined based on the number of additional entrances during control trials, divided by the minutes from the first entrance to the end of the trial. Each of these measures captures behavior at the level of the pair because individuals could not always be identified (as was done in Shephard et al., 2014). Testing at the pair, as opposed to individual level is justified, given that both parents care for the nestlings (Röell, 1978), and both must provide adequate food in order for nestlings to survive (Henderson and Hart, 1993). Therefore any impacts to fitness would be visible at the level of the pair.

A subset (16%) of trials were video coded by two people, and intercoder reliability was deemed to be excellent (ICC (1) = 0.959, p < 0.001).

We quantified breeding success in two ways: (1) the proportion of hatchlings that fledged in each nest, and (2) for nests that fledged chicks, we analyzed the average body condition of chicks within the brood at ringing. Body condition was calculated based on chicks’ residual deviation from the nestling population’s regression of weight against tarsus (e.g. Verhulst and Salomons, 2004), such that birds with a larger body weight than expected by their tarsus length were judged to be in relatively good condition. These two measures reflect the success of the pair’s foraging and nest defense efforts instead of their fertility. Also, since jackdaws have close to zero extra pair copulations (Henderson et al., 2000, Liebers and Peter, 1998) the chicks were assumed to belong to both parents.

### 2.4 Statistical analysis

We first determined how consistent boxes were in their neophobia score, control score, and provisioning rate across the two sampling periods with an Intra-Class Correlation (ICC) analysis (Nakagawa and Schielzeth, 2010). To assess whether or not control entrance times were related to object test entrances, we ran a Pearson’s product moment correlation test on the mean control and mean neophobia test times.

In order to determine the relationship between neophobia and provisioning rates we used a Cox proportional hazards regression model on birds’ nest entrance times during our tests and controls. This survival analysis examined the extent to which experimental condition, provisioning rate, test number (first or second test), order of conditions, time of day, days since the box hatched, hatch date, and all biologically meaningful interactions between these terms predicted entrance times. Each nest’s hatch date was defined in reference to the number of days since the first egg hatched within the population. Observations were clustered around box and around box colony to account for the non-independence of observations.

We conducted either Linear Mixed Models (LMM) or Generalized Linear Mixed Models (GLMM) on the factors that contributed each of the two measures of nest success (i.e. proportion of surviving chicks, and average nestling body condition), and the factors that influenced each hormone measure (i.e. baseline and stressed-induced). All models used the lme4 package (Bates et al., 2013) in R (R Development Team, 2015), when model outputs did not automatically give p-values, they were calculated from the t-value in the model summary. The nest success models investigated the influence of hatch date, neophobia score, control score, and provisioning rate, taking box colony into account as a random effect. The model on average nestling body condition also included the brood size as a fixed effect. The proportion of surviving chicks (no. fledged/no. hatched) was analyzed with a GLMM that had a binomial error structure and a logit link function. The mean body condition of chicks per nest was analyzed with an LMM. Hormone concentrations were log-transformed to create normal distributions before being analyzed with LMMs. Baseline hormone analyses assessed the impact of brood size, hatch date, provisioning rate, sex, weight in relation to siblings, and mean neophobia scores, controlling for the time of day, exact time of sampling from initial disturbance, body condition, and assigning nest box and nest box colony as random effects. Stressed-induced analyses contained the same set of variables, but also included baseline level as a covariate. All models were simplified through backwards stepwise elimination, based on changes in AIC values. Effects were retained if their exclusion increased AIC values by 2 or more. Once the final model was established, p values and effect sizes were calculated to be included in the text.

## 3 Results

### 3.1 Population nest success

Within the entire site, out of the 118 boxes, 72 hatched chicks and 53 successfully fledged young, (1.94 ± 0.63 fledglings per successful nest). Of the nineteen nests that failed during the 2013 season, 15 were tested for parental neophobia at least once prior to failure.

### 3.2 Individual consistency

Pairs were consistent in their entrance times during object test conditions (n = 44, ICC = 0.642, p < 0.001, CI = 0.43–0.79). Although this effect was strongly biased by the birds that did not return in either neophobia test — as removing them eliminated the effect of consistency (n = 29, ICC = 0.177, p = 0.172, CI = −0.19–0.50) — the fact that all birds returned for at least one of their two control trials indicates these non-returners were consistent in being particularly fearful of the object. In contrast, birds’ entrance times were not consistent during control conditions (n = 49, ICC = 0.174, CI = 0.11–0.43, P = 0.112), even though nest provisioning rate was consistent within pairs (n = 46, ICC = 0.321, CI = 0.04–0.56, P = 0.013). Additionally, the mean control and mean object test entrance times were correlated within nests (t = 2.28, df = 63, r = 0.276, CI = 0.03–0.49, P = 0.026).

### 3.3 Entrance times during experiments

**Fig. 2:**
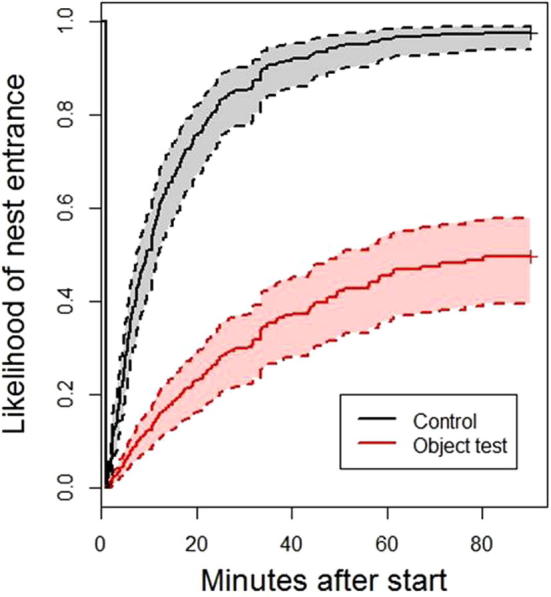
Likelihood of entering the nest. Inverted survival curves showing the likelihood over time that birds return to their nest boxes from the beginning of the trial. Dotted lines show confidence intervals.

Birds were slower to enter their nests when a novel object was present (Cox proportional hazards regression, n = 218 observations, 162 events, *B* = −1.77 ± 0.18, z = −9.89, P < 0.001; see Fig. 2) and birds with later hatch dates were slower to return to their nests, regardless of experimental condition (*B* = −0.10 ± 0.03, z = −2.67, P = 0.008) because there was no significant interaction between condition and hatch day. Additionally, birds with lower feeding rates took longer to return to their nest (*B* = −0.08 ± 0.02, z = 3.19, P = 0.001), but this effect was not specific to neophobia tests or control trials since there was no interaction between feeding rate and experimental condition (*B* = −0.01 ± 0.99, z = 1.26, P = 0.208). Therefore neophobia levels were not predictive of provisioning rates.

### 3.4 Individual nest success

Parents who had higher provisioning rates raised a greater proportion of their hatching young to fledging age (GLMM, n = 68 nests, Est = 0.095 ± 0.04, z = 2.22, P = 0.027; see Supplementary Table S1). Although larger broods had chicks that were of lesser quality on average (LMM, n = 53, Est = −11.01 ± 5.52, z = −2.00, P = 0.046), none of the other variables we measured, including parental neophobia scores, predicted the average body condition of chicks (Supplementary Table S2).

### 3.5 Hormone levels

**Fig. 3:**
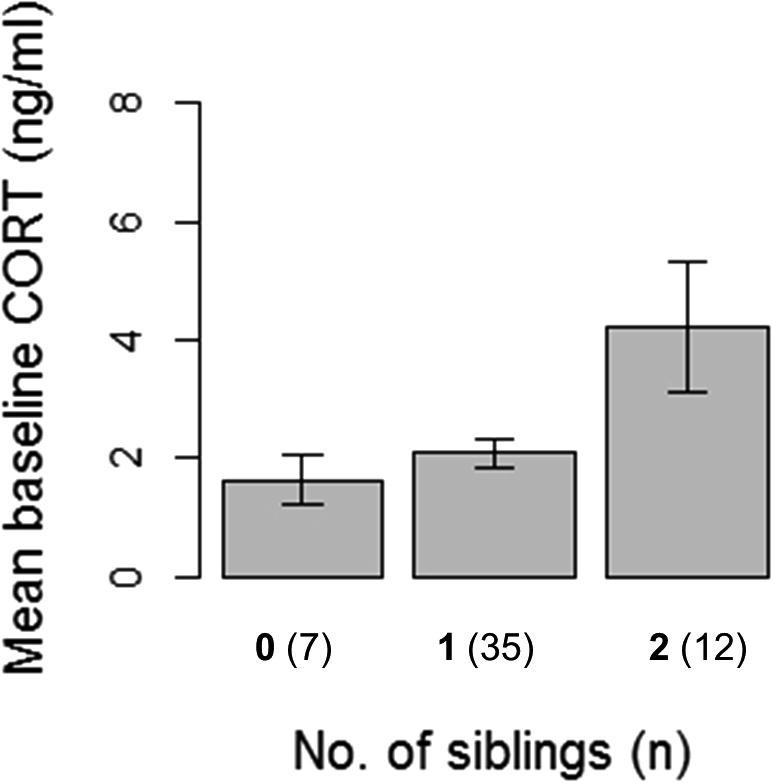
Baseline CORT by brood size. Means of raw baseline CORT levels for chicks within nests that have zero, one or two siblings present at the time of sampling. Error bars represent standard errors (SE’s) and numbers in parentheses indicate the number of individuals sampled from each brood size.

**Fig. 4:**
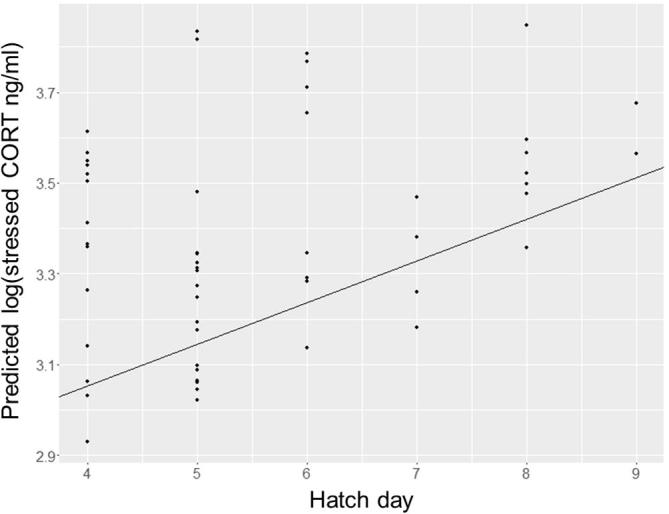
Predicted stress-induced CORT by hatch day. Predicted values were based on the output of an LMM, with stress-induced CORT on the logarithmic scale. Est. = 0.092 ± 0.04, z = 2.122, p = 0.034.

Baseline CORT values were higher for chicks with a greater number of siblings on ringing day (LMM, n = 57, Est = 0.392 ± 0.15, z = 2.63, P = 0.009; see Fig. 3, Supplementary Materials Table S3), but body condition, sex, parents’ neophobia score, and all other explanatory factors tested did not have an impact. Although baseline concentrations influenced individuals’ stress-induced levels of CORT (LMM, n = 55, Est = 0.062 ± 0.02, z = 2.70, P = 0.007) stress-induced values were not influenced by brood size. Instead, stress-induced levels were higher in chicks whose nest started later in the season (Est. = 0.092 ± 0.04, z = 2.122, P = 0.034; see Fig. 4, Supplementary Materials Table S4).

## 4 Discussion

**Fig. 5:**
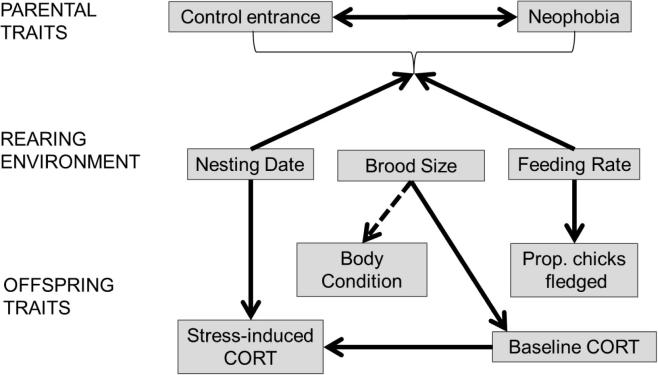
Summary of results. All boxes connected by arrows were statistically linked. Arrow type indicates the direction of the relationship: solid lines are positively correlated, dashed lines are negatively correlated. Boxes without arrows were not significantly related. Arrow direction does not imply causality, but the arrows point to the response variable in the analysis. Control entrance is the time at which birds entered their nests during controls; neophobia is the same measure during object neophobia trials.

Contrary to our predictions that parental neophobia levels would affect provisioning rates and the levels of developmental stress their offspring experience, we did not find correlations between neophobia, feeding rate, or offspring hormone levels. Although parents’ provisioning rate was the main predictor of chicks’ survival, as found in previous studies (Henderson and Hart, 1993), provisioning rate did not correlate with parental neophobia scores, nor with chicks’ body condition and stress levels. However, certain aspects of the rearing environment were associated with chicks’ stress hormone levels. For example, chicks from nests with larger broods had higher baseline CORT levels (Saino et al., 2003), and later hatching nests had higher CORT concentrations in response to handling stress, irrespective of chicks’ body condition. Since parents from later hatching nests also were slower to return in both experimental conditions, such a response may indicate that either these parents were more sensitive to nest disturbance, independent of novelty responses, or that they spent less time at their nests generally. Overall, the results reveal the importance of sibling competition and hatching date in contributing to natural variation in stress responses, but suggest that parents’ neophobia has no detectable influence on their reproductive success under the environmental conditions of this study. Fig. 5 provides a graphical illustration of the relationships between parental traits, rearing environments and offspring traits.

Although parents’ neophobia scores did not correlate with either the number or condition of their chicks, the scores themselves cannot be dismissed as noise. Neophobia scores and our provisioning rate measures were consistent across the season, with similar repeatability to that reported in studies on other species that have presented novel objects at nest boxes (Cole and Quinn, 2014). Given that individual variation across cognitive responses and traits may have important effects on fitness (Thornton and Lukas, 2012), one might expect this variation to have impacts on reproductive success. However, we found no impact of parental neophobia on either the percentage of hatching chicks that fledged per nest (similar to findings reported by Schuett et al., 2012), or the body condition of chicks. Given that jackdaws are known to be more neophobic than other passerine species, such as great tits (*Parus major*) (Greggor et al., 2016a), it may seem puzzling at first that we found no obvious costs or benefits to this distinctive trait.

Neophobia levels are suggested to impact fitness by increasing wariness and thus survival alongside predators and by helping with foraging among potentially dangerous resources (e.g. the dangerous niche hypothesis, Greenberg, 2003). This hypothesis relies on there being a high prevalence of predators, or poisonous prey, which could vary as environmental conditions change. Additionally, the same environmental conditions may impact the optimal level of neophobia differently depending on animals’ life stage. For instance, high neophobia increases survival in juvenile, predator naïve reef fish (Ferrari et al., 2015). Meanwhile higher parental neophobia is correlated with lower nest survival in great tits, supposedly because more neophobic individuals were less likely to challenge predators and defend their nests (Vrublevska et al., 2015). In this way, the same level of neophobia could have different costs and benefits depending on the life stage and the dangers of the environment, such that neophobia might be beneficial for juveniles who can flee predators but costly for adults when fleeing predators leaves their nests defenseless. Potentially, therefore, neophobia could impact jackdaw fitness or survival at a different life stage or time of year than what our breeding success measures capture.

One reason why neophobia did not impact reproductive success is because neophobia did not influence pairs’ combined provisioning rate. Since neophobic behavior involves the psychological appraisal of novelty (Greggor et al., 2015), neophobia would only aid in acquiring variable food if variability involved novel, not just patchy resources, or if food were often found near novel objects. Therefore reactions towards a novel object in a foraging context might be more relevant for fitness consequences than reactions in a nesting context. While object neophobia in corvids is repeatable when tested in the same context and time of year (Greggor et al., 2016b, Jolles et al., 2013), the consistency of individuals in the wild toward object neophobia tests in different contexts is rarely studied. Moreover, very little is known about how individual variation in object neophobia impacts natural feeding choices in the wild. Since we were unable to measure the extent to which single parents contributed to the pairs’ neophobia score and provisioning rate, it is possible that partners could compensate if one member of the pair was particularly neophobic, and therefore mask connections between neophobia and provisioning. However, as the reproductive output that we measured stemmed from pair-level success, the birds’ combined effort, and hence their combined neophobia, is likely to have the greatest bearing on fitness

Regardless of whether partner compensation was occurring, overall feeding rate did not predict either baseline or stress-induced CORT levels. This null result is surprising because nutritional deficits have been shown to impact CORT hormone levels in other corvids (Pravosudov and Kitaysky, 2006). Since higher feeding rates were associated with increased brood size (see Fig. 5), and increased brood size predicted elevated baseline CORT levels, the way food was allocated within the nest may explain why feeding rate did not impact CORT. The predictability of a food source, not just the total amount of food available can influence CORT expression (Buchanan et al., 2003). Having more siblings could decrease the predictability with which any one individual was fed. This effect seemed to impact all chicks within the brood similarly because we found no direct connection between baseline or stress-induced hormone levels and nestling body condition. An independence between baseline hormone levels and body condition contrasts with findings from studies of other birds (Müller et al., 2010, Rensel et al., 2011).

Since elevated baseline CORT encourages chicks to beg more often, long term increases in baseline CORT may act as an adaptive response to sibling competition, despite the costs that these hormones incur, such as later impacts on spatial memory (Kitaysky et al., 2003) and immune responses (Loiseau et al., 2008). Although higher levels of baseline CORT have been documented in experimentally enlarged clutches in other species (Saino et al., 2003) not all studies with brood manipulations or natural brood variation have found such an effect (Bize et al., 2010, Brewer et al., 2010, Müller et al., 2010). These differences between species in the effect of brood size on CORT cannot be explained by differences in hatching asynchrony. Even though it is unclear why larger broods of jackdaws have higher baseline CORT when other species may not, there are likely to be long-term effects of such sibling competition on individuals from larger broods.

Rearing conditions also influenced chicks’ stress-induced stress levels, as later hatching nests had higher stress-induced CORT values. There are two potential explanations for this effect, namely that late season chicks may have had worse parents, or that they may have experienced a different surrounding environment than early breeders. We found that parents from later season nests were slower to return in both control and object test conditions, which could mean that later season parents were more sensitive to disturbances such as a trial setup, or that they generally visited less often. Although nests that were slower to return in test and control conditions were also more likely to have lower provisioning rates, provisioning rate itself did not directly predict stress-induced CORT levels. Instead, later season jackdaws’ reluctance to return to the nest might have been indicative of lower levels of nest attendance. Reductions in nest attendance have been shown to alter stress hormone physiology in nestling Florida scrub-jays (*Aphelocoma coerulescens*), which has been suggested to be the result of the social stress of separation from the mother (Rensel et al., 2010). Therefore the parenting of late breeders’ might be to blame for the increases in stress-induced CORT we found.

Alternatively, the hormonal difference might not be due to the characteristics of late breeding parents, but to some type of external stress that impacts late nests disproportionately. Overall, later breeding individuals in many species produce smaller or poorer quality clutches (e.g. Hochachka, 1990, Winkler and Allen, 1996), but whether their poor performance is a result of individual quality is unclear because timing and quality are often intertwined (Verhulst and Nilsson, 2008). Although later nests fledged a similar number and quality of chicks, their elevated stress-induced hormone levels could indicate that late hatching individuals might be on a different developmental trajectory that predisposes them to be more responsive to acute stressors.

Although we found no impact of parental neophobia on offspring CORT levels, the variation in baseline and stress-induced CORT that we detected among nestlings could potentially contribute to downstream variation in their stress responses as adults. Since experiencing elevated levels of CORT during development may modify the negative feedback loops of stress hormone expression (Romero, 2004, Zimmer et al., 2013), the impact of sibling competition and later hatch date may determine how individuals cope with future stressors. Moreover, since the expression of neophobia and CORT are thought to be linked within individuals (Bebus et al., 2015), and there is evidence that experimentally administering CORT during development increases neophobia later in life, at least in males (Spencer and Verhulst, 2007), differences in the rearing environment might also contribute to variation in neophobia in adulthood. Testing whether or not, for example, chicks in larger broods show differing levels of neophobia as adults could help determine the long term consequences of early life stress and help explain why we see variation in neophobia without clear fitness consequences.

Investigating the development of individual differences in stress physiology helps explain some of the variation in cognitive traits, and stress responses seen in the wild. Neophobia, provisioning rates and CORT were not connected in this study. If this disconnect is true for a number of species, then perhaps we need to re-examine under what ecological conditions neophobia should be favored. Future research needs to determine whether neophobia is not predictive of the quality of rearing environment across a greater diversity of environmental conditions when food is scarce and innovation could be helpful. Also, assessing the fitness consequences of neophobia at other times of year could help inform where neophobia might benefit individuals. Without such assessments the ecological consequences of individual variation in traits such as neophobia will remain elusive.

## Funding

This work was supported by the Gates Cambridge Trust to ALG and two separate BBSRC David Phillips Fellowships to KAS and AT (BB/L002264/1 and BB/H021817/1).
