# Supplementary material for: Wild jackdaws’ reproductive success and their offspring’s stress hormones are connected to provisioning rate and brood size, not to parental neophobia
Source: Gen Comp Endocrinol. 2017 Mar 1;243:70–7. doi: 10.1016/j.ygcen.2016.11.006 (PMC5325159; doi:10.1016/j.ygcen.2016.11.006)
Supplement: Supplementary data 1 [file mmc1.docx]

Variation in stress hormones and reproductive success in wild jackdaws (*Corvus monedula*) is connected to brood size and provisioning rate, not to neophobia

Alison L. Greggor^1,2^, Karen A. Spencer^3^, Nicola S. Clayton^1^, Alex Thornton^4^

^1^Department of Psychology, University of Cambridge

^2^Department of Biological Sciences, Dartmouth College

^3^School of Psychology and Neuroscience, University of St. Andrews

^4^Department of Biosciences, University of Exeter, Penryn

Supplementary Materials


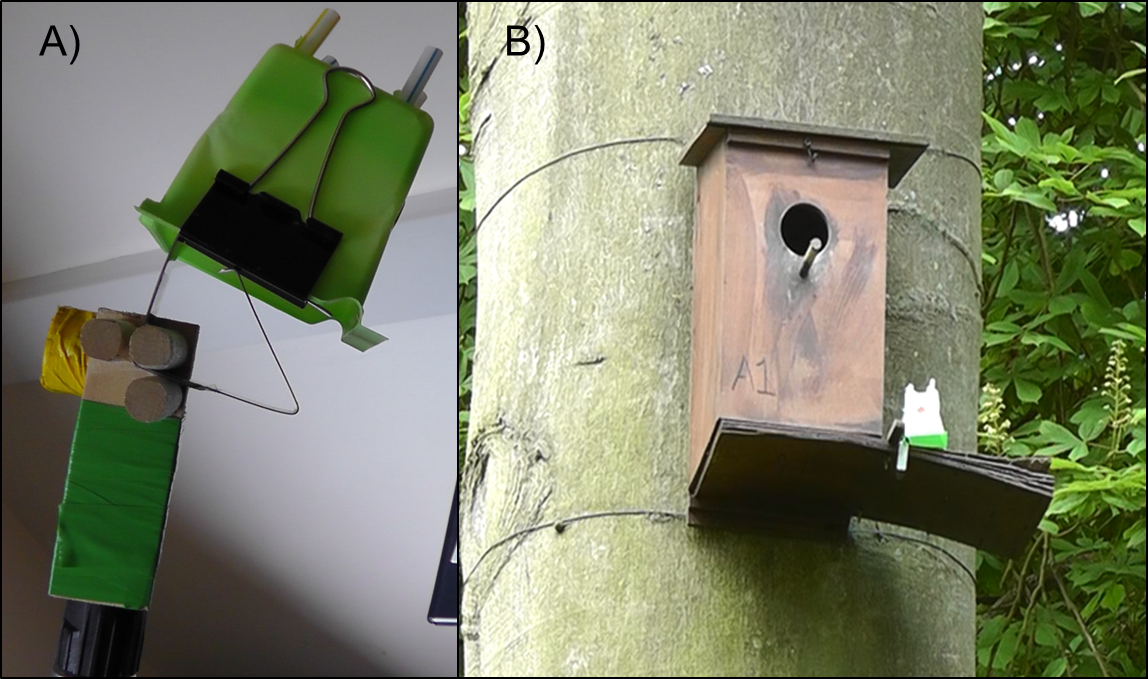


**Figure S2.** Experimental setup. An extendable pole with an attachment (A) was used to place objects on box platforms (B).

**Table S1**. **Factors impacting proportion of chicks fledged.** Box colony was included as a random effect. Terms were dropped if their exclusion increased the AIC value by less than two. The bold model is the minimal model.

| Proportion of chicks surviving GLMM, n=68 | ∆AIC |
| --- | --- |
| Num_fledge/Num_hatch~ Feed_Rate+ Control + Hatch_day + Test + + Feed_Rate:Hatch_day + Test:Hatch_day | 0.0 |
| Num_fledge/Num_hatch~ Feed_Rate+ Control + Hatch_day + Test + + Feed_Rate:Hatch_day | - 0.6 |
| Num_fledge/Num_hatch~ Feed_Rate+ Control + Hatch_day + Test | - 1.1 |
| Num_fledge/Num_hatch~ Feed_Rate+ Control + Hatch_day | - 1.7 |
| Num_fledge/Num_hatch~ Feed_Rate + Control | - 1.0 |
| **Num_fledge/Num_hatch~ Feed_Rate** | **- 0.0** |
| Num_fledge/Num_hatch~ 1 | + 3.0 |

**Table S2**. **Factors impacting mean body condition of fledging chicks per nest**. Individual body condition was determined based on their deviation from the regression of weight against tarsus. Box colony was included as a random effect. Terms were dropped if their exclusion increased the AIC value by less than two. The bold model is the minimal model.

| Mean body condition of nest GLMM, n=53 | ∆AIC |
| --- | --- |
| Condition~ Num_fledge + Test +Feed_Rate + Num_fledge:Feed_Rate + Hatch_day + Test:Hatch_day + Control | 0.0 |
| Condition~ Num_fledge + Test +Feed_Rate + Num_fledge:Feed_Rate + Hatch_day + Test:Hatch_day | - 1.8 |
| Condition~ Num_fledge + Test +Feed_Rate + Num_fledge:Feed_Rate + Hatch_day | - 1.2 |
| Condition~ Num_fledge + Test +Feed_Rate + Num_fledge:Feed_Rate | - 1.3 |
| Condition~ Num_fledge + Test +Feed_Rate | + 0.9 |
| Condition~ Num_fledge + Test | - 1.5 |
| **Condition~ Num_fledge** | **- 0.6** |
| Condition~ 1 | + 2.0 |

**Table S3**. Factors influencing baseline CORT levels. Box colony and box ID were included as random effects. Interactions are denoted with a “:”. The bold model is the minimal model.

| LMM, n=57 | ∆AIC |
| --- | --- |
| log(Baseline)~ Brood_size + Blood_sample_time + Chick_quality + Brood_size:Chick_quality + Hatch_day + Feed_rate + Neophobia_score + Time_of_day + Sex + Comparative_weight + Control_score | 0.0 |
| log(Baseline)~ Brood_size + Blood_sample_time + Chick_quality + Brood_size:Chick_quality + Hatch_day + Feed_rate + Neophobia_score + Time_of_day + Sex + Comparative_weight | - 2.0 |
| log(Baseline)~ Brood_size + Blood_sample_time + Chick_quality + Brood_size:Chick_quality + Hatch_day + Feed_rate + Neophobia_score + Time_of_day + Sex | - 2.0 |
| log(Baseline)~ Brood_size + Blood_sample_time + Chick_quality + Brood_size:Chick_quality + Hatch_day + Feed_rate + Neophobia_score + Time_of_day | - 1.9 |
| log(Baseline)~ Brood_size + Blood_sample_time + Chick_quality + Brood_size:Chick_quality + Hatch_day + Feed_rate + Neophobia_score | - 1.9 |
| log(Baseline)~ Brood_size + Blood_sample_time + Chick_quality + Brood_size:Chick_quality + Hatch_day + Feed_rate | - 1.6 |
| log(Baseline)~ Brood_size + Blood_sample_time + Chick_quality + Brood_size:Chick_quality + Hatch_day | - 1.8 |
| log(Baseline)~ Brood_size + Blood_sample_time + Chick_quality + Brood_size:Chick_quality | - 0.7 |
| log(Baseline)~ Brood_size + Blood_sample_time + Chick_quality | - 0.1 |
| log(Baseline)~ Brood_size + Blood_sample_time | - 1.8 |
| **log(Baseline)~ Brood_size** | **- 1.0** |
| log(Baseline)~ 1 | + 4.7 |

**Table S4**. Factors influencing stress-induced CORT levels. Box colony and box ID were included as random effects. Interactions are denoted with a “:”. The bold model is the minimal model.

| LMM, n=56 | ∆AIC |
| --- | --- |
| log(Stress-induced)~ Baseline + Hatch_day + Feed_rate + Control_score + Neophobia_score + Chick_quality + Comparative_weight + Time_of_day + Brood_size + Sex + Blood_sample_time | 0.0 |
| log(Stress-induced)~ Baseline + Hatch_day + Feed_rate + Control_score + Neophobia_score + Chick_quality + Comparative_weight + Time_of_day + Brood_size + Sex | - 2.0 |
| log(Stress-induced)~ Baseline + Hatch_day + Feed_rate + Control_score + Neophobia_score + Chick_quality + Comparative_weight + Time_of_day + Brood_size | - 2.0 |
| log(Stress-induced)~ Baseline + Hatch_day + Feed_rate + Control_score + Neophobia_score + Chick_quality + Comparative_weight + Time_of_day | - 1.3 |
| log(Stress-induced)~ Baseline + Hatch_day + Feed_rate + Control_score + Neophobia_score + Chick_quality + Comparative_weight | - 1.6 |
| log(Stress-induced)~ Baseline + Hatch_day + Feed_rate + Control_score + Neophobia_score + Chick_quality | - 0.3 |
| log(Stress-induced)~ Baseline + Hatch_day + Feed_rate + Control_score + Neophobia_score + | - 2.0 |
| log(Stress-induced)~ Baseline + Hatch_day + Feed_rate + Control_score | - 0.1 |
| log(Stress-induced)~ Baseline + Hatch_day + Feed_rate | - 1.4 |
| **log(Stress-induced)~ Baseline + Hatch_day** | **- 0.4** |
| log(Stress-induced)~ Baseline | + 2.5 |
